# Injury Incidence and Its Characteristics in Korean Youth and Collegiate Taekwondo Sparring Athletes: A Retrospective Study

**DOI:** 10.3390/ijerph20085528

**Published:** 2023-04-17

**Authors:** Mi-ock Han, Nam-kyung Lee, Hyung-pil Jun

**Affiliations:** 1 Department of Physical Education, Dong-A University, Busan 49315, Republic of Korea; 2 International Olympic Committee Research Centre Korea, Seoul 03722, Republic of Korea

**Keywords:** Taekwondo athletes, injury characteristics, injury incidence rate, 1000 athletic exposures

## Abstract

This study aimed to identify the incidence of injury and its characteristics in Korean youth and collegiate Taekwondo athletes during 2021 and to provide a suggestion regarding injury incidence. A total of 183 athletes (95 youth and 88 collegiate athletes) who were registered with the Korea Taekwondo Association (KTA) participated. The research was based on the injury questionnaire developed by the International Olympic Committee (IOC). The questionnaire consists of a total of seven items, including four items related to demographic characteristics and three items related to injuries (locations of injury, types of injury, and causes of injury). A frequency analysis was performed to identify the injury characteristics. Additionally, the injury incidence rate (IIR) was calculated based on 1000 athletic exposures (AEs) during 2021. The IIRs for one recent year (2021) showed 4.43/1000 AEs and 3.13/1000 AEs in youth and collegiate Taekwondo athletes, respectively. The frequency analysis showed that finger (youth: 17.3%, collegiate: 14.6%), contusion (youth: 25.3%, collegiate: 23.8%), and contact with other athletes (youth: 57.6%, collegiate: 54.4%) ranked the highest in terms of the locations of injury, types of injury, and causes of injury, respectively. A continuing injury tracking system can play a key role in accumulating big data for identifying risk factors and developing interventions to reduce injury in Taekwondo sparring.

## 1. Introduction

To date, the effects of Taekwondo training that have been studied have shown positive effects on the physical development and improvement of physical abilities of children, adolescents, and adults [1,2,3]. In particular, physical fitness factors (e.g., muscle strength, agility, and balance) [4,5] and physiological factors (e.g., obesity, high blood pressure, and cardiovascular disease) [6,7] showed effective improvement, and many people participate in Taekwondo without restrictions in terms of age or gender. However, these studies have been conducted on ordinary people, not elite Taekwondo athletes. Since these individuals train at a recreational level (low-intensity), it is difficult to transpose these positive effects onto elite Taekwondo athletes.

In Korea, in order to train elite Taekwondo sparring athletes, various training systems such as team training and professional coaching are utilized from their childhood [8]. Young Taekwondo sparring athletes undergo daily repeated high-intensity training (tactics, physical fitness, and techniques) for a long time [9,10,11]. Participation in high-intensity sports is accompanied by an inherent risk of musculoskeletal injuries [12]. In the case of adolescents in particular, this may interfere with inadequate neuromuscular function and the physical ability of the musculoskeletal system compared to increased bone growth rate [13]. In addition, they are exposed to a risk of injury for various other reasons [9,10,11]. One of these reasons is a rule whereby sparring athletes earn points by striking opposing athletes, so injuries due to contact in sparring are higher than among demonstration and poomsae athletes (who engage in a series of attach and defense forms without contact) [14].

Recent studies on sparring in Taekwondo have reported 27.59/1000 athletic exposures (AEs) for youth athletes and 19.96/1000 AEs for adults during competitions [15], and the lower extremity is the most commonly injured body part [16]. In order to minimize the incidence of injury in Taekwondo, the application of new technologies and big changes to the rules have been implemented in all official Taekwondo competitions by World Taekwondo, including changes in the application of electronic protective gears, the synchronizing of an electronic headgear system, and the scoring system, as well. During sparring in Taekwondo, it is well-known that ‘sparring’ skills consist of offensive and defensive blocks, punches, and kicks, and these skills are used to strike opposing athletes in order to score points. On the other hand, many athletes face unexpected skills/moments as a result of anomalous kicks, which are modified versions of traditional kicks that continuously result in injuries [17]. To protect athletes from the accumulative impact of striking by punching and kicking, it is mandatory to wear protective equipment [18]. However, it is difficult to prevent injuries when wearing the same protective gear, regardless of the weight class [19]. Generally, each Taekwondo athlete in ‘sparring’ competitions predicts the opponent’s movements and performs his/her skills. For skills that require excessive movements in the joints (hip, knee, and ankle) of the lower extremities, in particular [20,21], instantaneous rotation and force are applied to each lower extremity segment during these continuous movements in practice/competitions. These repeated motions generate tensional force, which causes degenerative changes and functional deterioration in soft tissues such as muscles, ligaments, and capsules [8].

As was mentioned above, athletes suffer from injuries continuously and research that aims to identify the injury incidence rate is ongoing [15,16]. However, to date, reports on injuries in both Korean youth and collegiate Taekwondo athletes are still insufficient. In addition, there are currently more than 9.6 million (5 million youth and 4.5 million adult) Taekwondo athletes who hold a black belt and are registered at Kukkiwon worldwide, and they participate in many competitions hosted by the Korea Taekwondo Association (KTA) every year [22]. In order to minimize the incidence of injury for this athletic population, researchers need to find out which factors are related to the incidence of injury.

Therefore, the purpose of this study was to identify the incidence of injury and its characteristics in youth and collegiate Taekwondo athletes for a year, retrospectively, and to provide a suggestion regarding injury incidence. The hypotheses were: (1) injury incidence rates (IIR)/1000 athlete exposures (AEs) are higher in youth athletes, (2) ankles are more prone to injury than other body parts, (3) sprain is the most common injury type, and (4) contact with other athletes is the most common cause of injury.

## 2. Materials and Methods

### 2.1. Participants

To recruit eligible participants, our research team visited 11 junior varsity, 8 varsity and 4 university teams which were located in the southern region of South Korea, and instructed their coaches and certified athletic trainers (ATC) to screen their eligibility. This screening process for their eligibility was conducted based on inclusion/exclusion criteria. The inclusion criteria were: (1) athletes who were registered in the Korea Taekwondo Association (KTA) in 2021, (2) youth athletes (between 12 and 18 years old), and (3) collegiate athletes (over 19 years old). The exclusion criteria were individuals who: (1) had never participated in competitions organized by their regional Taekwondo association or the KTA, and (2) were not enrolled in junior varsity, varsity, or university teams. After the screening, a total of 183 athletes (95 youth athletes and 88 collegiate athletes) participated in this study. Prior to participation in this study, each athlete signed their informed consent after all procedures were explained. In the case of youth athletes, they were included only after obtaining parental consent as vulnerable research subjects (juveniles) according to the Research Ethics Act (Article 16). This study was approved by the Institutional Review Board at Dong-A University (IRB No. 2-1040709-AB-N-01-202109-HR-072-08). All authors declare that the research was carried out following the rules of the Declaration of Helsinki of 1975, revised in 2013.

### 2.2. Procedure

Once each participant had signed an informed consent form, he/she answered the questionnaire regarding his/her demographic characteristics and previous and current injuries to investigate the characteristics of their injuries from January to December in 2021. Each athlete was instructed to recall his or her previous injuries that were diagnosed by a doctor based on their memory as much as they could, and they were allowed to make multiple selections. A researcher sat with each athlete and explained each question of the questionnaire one by one, so that the athlete could answer correctly and to minimize errors from misunderstanding. After the completion of the questionnaire, each participant’s anthropometric data (height, weight, body mass index, and body fat) were measured using an Accuniq BC720 (Jawon Medical, Daejeon, Republic of Korea) impedance body composition analyzer. In order to measure accurate anthropometric data, an examiner instructed each participant according to the manufacturer’s manual. Each athlete was informed about contraindications before measurement, including (1) not to drink any liquid at least 2 h before measurement, (2) no vigorous exercise before measurement, (3) no meals before measurement, (4) no wearing jewelry during measurement, and (5) no movement during measurement.

### 2.3. Questionnaire

The questionnaire was based on the injury questionnaire developed by the International Olympic Committee (IOC) [23]. The questionnaire consists of a total of 7 items, including 4 items related to demographic characteristics (age, sex, athletic career, and practice time (hours/day)) and 3 items related to injuries (locations of injury, types of injury, and causes of injury). Athletic career was defined as the time (in months) from the first registration with the KTA as a sparring athlete to the date of their participation in this study. Practice injury indicated the number of injuries during practice and was equal to the number of injury locations. Practice time (hours) represented the sum of daily training time in hours for each gender and group. Additionally, injury was defined as newly diagnosed, recurring musculoskeletal complaints, and other orthopedic conditions sustained during practice in 2021.

### 2.4. Statistical Analysis

A frequency analysis was performed to identify injury characteristics using SPSS 27 (IBM Corporation, Armonk, NY, USA). Based on the data from the questionnaire, the injury incidence rate (IIR) was calculated based on athletic exposure (AEs) during 2021 using the following formula:

$$\frac{\mathrm{new}\;\mathrm{injuries}\;\mathrm{during}\;\mathrm{the}\;\mathrm{year}}{\left(\mathrm{participants}\;\mathrm{in}\;\mathrm{the}\;\mathrm{company}\right)\left(\mathrm{annual}\;\mathrm{hours}\;\mathrm{participation}\;\mathrm{in}\;\mathrm{practice}\right)\;}\times 1000$$

The 95% Confidence Interval (95% CI) of the IRR was calculated based on the total number of practice injurys, total of practice time, standard error, and IIR using the following formula:

$$\left(\frac{\mathrm{IIR}}{1000}\;\pm 1.96\mathrm{SE}\right)\times 1000$$

## 3. Results

### 3.1. Injury Incidence Rates (IIR) of Youth and Collegiate Taekwondo Athletes

A total of 550 injuries was recorded during 124,176 AEs among the 95 registered youth Taekwondo sparring athletes, giving us a result of 4.43/1000 AEs. Additionally, a total of 536 injuries was recorded across 171,288 AEs among 88 registered collegiate Taekwondo sparring athletes, giving us a result of 3.13/1000 AEs. Table 1 shows the characteristics of the athletes and injuries during practice by group and sex.

### 3.2. Injury Characteristics of Youth and Collegiate Taekwondo Athletes

The most common locations for injuries are reported in order in Table 2. In particular, the finger was the most common location of injury in both the youth (17.3%) and collegiate (14.6%) Taekwondo sparring groups. Contusion was the most common type of injury in both the youth (25.3%) and collegiate (23.8%) Taekwondo sparring groups, and the other types of injury are shown in Table 3. The most common cause of injury was contact with other athletes in both the youth (57.6%) and collegiate (54.4%) Taekwondo sparring groups, but the order of the most common causes of injury are slightly different between the two groups, as is shown in Table 4.

## 4. Discussion

To accomplish the purpose of this study, this retrospective study was performed for one recent year (January to December 2021) using an injury questionnaire survey, and the data were analyzed by group. For a period of 12 months in 2021, the youth Taekwondo sparring athletes were more prone to injury compared to the collegiate Taekwondo sparring athletes. The most common location of injury was a finger, regardless of group. In both groups, contusion was the most common type of injury. Moreover, contact with other athletes was the most common encountered cause of injury in both of the two groups. 

In Taekwondo sparring, youth athletes exhibited 4.43/1000AEs of IIR while collegiate athletes showed 3.13/1000AEs. This result is inconsistent with the results of previous studies, which found that adult Taekwondo elite athletes experienced more injuries than youth Taekwondo elite athletes in both practice and competition situations [24]. Based on this current study, Korean youth Taekwondo athletes have an average exercise time of 196.2 min per day for six days per week, which is similar to that of adult Taekwondo elite athletes [25]. High intensity training is considered to be one of the potential risk factors for youth and adult athletes’ injuries due to overtraining or overuse. In particular, this seems to be related to the reason why youth athletes are more prone to sustain injury, as adolescents have an incomplete musculoskeletal system compared to adults during their growth phase [8,25]. In addition, according to the results of the most recent previous study [26], the most common cause of sparring players’ injuries is contact with their opponents; this is exactly the same result as in this study. We emphasized sparring skills in the introduction; striking the opposing athlete to score points occurs continuously during combat. In this type of sport, striking includes kicking and punching (contact with other athletes) which could be an external impact cause of inflammation in body tissues, activating pain receptors in the body and increasing muscle tension, resulting in muscle fiber adhesion, decreased neuromuscular control, and muscle imbalance [27,28,29,30].

The most commonly injured body part was fingers in both youth and collegiate Taekwondo athletes, followed by ankle, knee, and foot/toes, respectively, in youth Taekwondo athletes and foot/toes, ankle, and thigh, respectively, in collegiate Taekwondo athletes. These results are consistent with previous studies showing that the highest incidence of injury in the upper extremities of Taekwondo athletes was in the fingers (42.8%) [19,21,31], and is partially consistent with previous studies in which lower extremity injuries (59.7–72.4%) such as ankles (15.4–30.6%), foot/toes (5.8–27.0%), knees (10.9–20.1%), and thighs (7.1–11.7%) were higher than those of the upper extremities (11.2–15.2%) [32,33,34]. In order to simultaneously defend oneself from an opponent’s attack and succeed in one’s own attack in the meantime, an accurate blocking action in preparation for the attack is essential during both practices and competitions [19]. However, most Taekwondo sparring athletes defend/block kicks without making a complete fist, and they acquire more injuries during blocking and defending movements than when attacking with punching during either practices or competitions [19]. In addition, martial arts (e.g., Taekwondo, boxing, and Karate) that include striking techniques cause the highest number of injuries despite the use of dedicated protective equipment to prevent hand injuries [35]. Hand injuries occur more frequently during competition, but are still underestimated and lead to significant long-term losses of function and/or deformities [35]. Since sparring in Taekwondo includes a high frequency of kick attacks, we speculated that blocking techniques to block the kicks and the improvement of hand protection equipment are essential for an injury prevention strategy.

In the lower extremity, the ankle was the most common injured site in this study and previous studies. This result could possibly be explained by the movement in Taekwondo sparring, for example when performing kicking techniques (e.g., cut kicking, round house kicking, back kicking, monkey kicking, etc.) while simultaneously changing direction rapidly with footsteps. Even during practice, this requires a lot of kicking. In other words, since sparring in Taekwondo includes a high frequency of kick attacks, the opponent needs to block the kicks continuously during practice and competition. These repeated situations may contribute to the causes of injury to the peripheral body segments of upper and lower extremities, such as the hand and ankle, respectively.

The types of injury were not significantly different between the youth and collegiate Taekwondo athletes. This result is supported by those of previous studies showing that contusions and sprains were the most common types of injuries in Taekwondo sparring [11,21,32]. The wearing of protective gear that covers the head, torso, arm, hand, shin, foot, teeth, and groin is mandatory during sparring. However, it could be interpreted that the role of absorbing the shock generated by striking is insufficient because the difference between the athlete’s body composition (height, weight, etc.) is not considered and compensated for by weight class [36]. In addition, it has been reported that the incidence of injuries is high when putting one’s foot down after kicking an opponent [33]. This might be explained by a lack of balance and/or concentration, or muscle fatigue during competitions [37]. In general, collegiate athletes have stronger structural characteristics than youth athletes. It has been reported that the risk of musculoskeletal injury is higher among youth athletes due to changes in their physiological characteristics [38].

Regarding the cause of injury, contact with other athletes is the most common mechanism. The results of this study are consistent with those of previous studies that stated that athletes could experience injury more often due to a situation where an athlete has to perform his/her techniques despite recognizing the performance of the opponent’s technique [8,24]. For youth athletes, the role of a coach in training seems to be an important contributor to injury prevention. In particular, the prevention of injuries resulting from negligence, recurrence, and overuse should focus on the correct training sequences and functional training, including kicking skills and simulation [32]. In addition, it has been reported that training during rapid weight loss may lead to overtraining or overuse and may have direct or indirect negative effects that can cause injury [39]. Regarding the countermeasures against contact with other athletes, which is the most common cause of injuries, it is believed that the blocking technique against striking should be improved [19]. In addition to contact with other athletes, other causes of injury included inattention, reoccurrence, overuse, and overtraining, sequentially. In a previous study including elite Taekwondo sparring athletes, the distribution of mechanisms of injury demonstrated a very similar trend [24].

This present study has the strength of being an epidemiological study for Taekwondo sparring athletes. This study was designed as an initial step in an injury prevention model according to Van Mechelen et al. (1992) and Finch (2006). Van Mechelen et al. presented the first injury prevention model, which has four steps: (1) establish the extent of the problem, (2) establish the etiology and mechanisms of injury, (3) introduce preventive measures, and (4) assess their effectiveness by repeating state 1 [40]. Finch updated the model by Van Mechelen due to a limitation of the first model. Her proposed framework is entitled “Translating research into injury prevention practice (TRIPP)”, and is for research leading to real-world sports injury prevention. It consists of six stages, including (1) injury surveillance, (2) establish etiology and mechanisms of injury, (3) develop preventive measures, (4) ideal conditions/scientific evaluation, (5) describe intervention context to inform implementation strategies, and (6) evaluate the effectiveness of preventive measures in the implementation context [41]. Based on these models, this study was conducted as stage 1, which is necessary for injury prevention research, so we could continue to the next steps to make a scientific evaluation for injury prevention. In addition, the response rates of Taekwondo athletes to the questionnaire were 100% of the total athletes; this represents the importance of this topic area in Taekwondo. Additionally, this study provided injury characteristics during practice in Taekwondo sparring, so that coaches and trainers can understand and implement preventative training or practice to reduce injury.

On the other hand, this study has limitations. The first is a potential recall bias. Taekwondo sparring athletes were asked to recall any injury that they had suffered during practice over one year. The second was that competitions were restricted during 2021 due to the COVID-19 pandemic. Not only were Taekwondo sparring athletes unable to participate in competitions, but neither were any other sports athletes, according to directions from the Korean government. Therefore, data were accessed only for practice and not competition. In addition, participants were not equally distributed by gender, athletic career, and age within each group. The results and conclusions from this study cannot be generalized for all Taekwondo sparring athletes in Korea because this study investigated a limited region of Korea.

## 5. Conclusions

In summary, youth Korean Taekwondo sparring athletes demonstrated a higher injury incidence rate than collegiate Korean Taekwondo sparring athletes during 2021. However, the injury characteristics showed similarities between youth and collegiate groups, as follows: (1) the most common location of injury was the finger, (2) contusion was the most common type of injury, and (3) contact with other athlete was the most commonly encountered cause of injury. Based on the results of this study, a continuing injury tracking system can have a key role in accumulating big data for identifying risk factors and developing interventions to reduce injury in Taekwondo sparring. 

## Figures and Tables

**Table 1:** Practice information and injury incidence rates of youth and collegiate Taekwondo athletes (M ± SD).

Variables	Youth Taekwondo Athletes			Collegiate Taekwondo Athletes		
	Male (*n* = 59, 62.1%)	Female (*n* = 36, 37.9%)	Total (*n* = 95, 100%)	Male (*n* = 70, 79.5%)	Female (*n* = 18, 20.5%)	Total (*n* = 88, 100%)
Athletic Career (months)	55.17 ± 23.59	43.69 ± 23.40	49.76 ± 25.11	122.79 ± 30.69	114.00 ± 31.41	120.99 ± 30.86
Practice Injury	316	234	550	428	108	536
Practice Time (hours/day)	257.5	140.5	398	471	78	549
AEs During Practice (hours)	80,340	43,836	124,176	146,952	24,336	171,288
IIR/1000AEs (95% CI)	3.93 (3.50–4.36)	5.34 (4.66–6.02)	4.43 (4.06–4.80)	2.91 (2.63–3.19)	4.44 (3.60–5.28)	3.13 (2.87–3.39)

Note: Values are shown as means ± SD; BMI: body mass index; IIR: injury incidence rates; AEs: athlete exposures; 95% CI: 95% Confidence Interval.

**Table 2:** Number of injuries in youth and collegiate Taekwondo athletes according to body location.

Body Part	Youth Athletes	Collegiate Athletes	Total
Head	34 (6.2)	32 (6.0)	66 (6.1)
Face	22 (4.0)	24 (4.5)	46 (4.2)
Neck/Cervical	1 (0.2)	3 (0.6)	4 (0.4)
Shoulder/Clavicle	2 (0.4)	5 (0.9)	7 (0.6)
Sternum/Ribs	4 (0.7)	1 (0.2)	5 (0.5)
Back/Thoracic	0 (0)	2 (0.4)	2 (0.2)
Waist/Lumbar	35 (6.4)	37 (6.9)	72 (6.6)
Abdomen	1 (0.2)	0 (0)	1 (0.1)
Humerus	9 (1.6)	6 (1.1)	15 (1.4)
Elbow	9 (1.6)	15 (2.8)	24 (2.2)
Forearm	14 (2.5)	4 (0.7)	18 (1.7)
Wrist	29 (5.3)	33 (6.2)	62 (5.7)
Finger	95 (17.3)	78 (14.6)	173 (15.9)
Pelvis/Sacrum/Buttock	7 (1.3)	13 (2.4)	20 (1.8)
Hip	2 (0.4)	2 (0.4)	4 (0.4)
Groin	2 (0.4)	3 (0.6)	5 (0.5)
Thigh	19 (3.5)	46 (8.6)	65 (6.0)
Knee	70 (12.7)	41 (7.6)	111 (10.2)
Tibia	23 (4.2)	33 (6.2)	56 (5.2)
Ankle	94 (17.1)	71 (13.2)	165 (15.2)
Achilles Tendon	9 (1.6)	9 (1.7)	18 (1.7)
Foot/Toe	69 (12.5)	78 (14.5)	147 (13.5)
Total	550 (100)	536 (100)	1086 (100)

Note: Values are shown as n (%).

**Table 3:** Types of injury in youth and collegiate Taekwondo athletes.

Types	Youth Athletes	Collegiate Athletes	Total
Contusion	139 (25.3)	131 (23.8)	270 (24.5)
Sprain	117 (21.3)	111 (20.2)	228 (20.7)
Fracture	75 (13.6)	78 (14.2)	153 (13.9)
Strain/Muscle Tear	30 (5.5)	51 (9.3)	81 (7.4)
Ligamentous Rupture	46 (8.4)	46 (8.4)	92 (8.4)
Nerve Injury/Spine Cord Injury	16 (2.9)	25 (4.5)	41 (3.8)
Stress Fracture	8 (1.5)	22 (4.0)	30 (2.7)
Other Bone Injuries	50 (9.1)	15 (2.7)	65 (5.9)
Fasciitis	3 (0.5)	11 (2.0)	14 (1.3)
Tendinitis/Tendinopathy	12 (2.2)	10 (1.8)	22 (2.0)
Other	4 (0.7)	10 (1.8)	14 (1.3)
Dislocation/Subluxation	3 (0.5)	9 (1.6)	12 (1.1)
Laceration/Abrasion	16 (2.9)	9 (1.6)	25 (2.3)
Meniscus	10 (1.8)	8 (1.5)	18 (1.6)
Muscle Spasm	9 (1.6)	7 (1.3)	16 (1.5)
Osteoarthritis/Synovial Capsulitis/Bursitis	7 (1.3)	3 (0.5)	10 (0.9)
Cerebral Concussion	3 (0.5)	2 (0.4)	5 (0.5)
Tendon Rupture	0 (0)	2 (0.4)	2 (0.1)
Tooth Damage	2 (0.4)	0 (0)	2 (0.1)
Total	550 (100)	550 (100)	1100 (100)

Note: Values are shown as n (%).

**Table 4:** Causes of injury in youth and collegiate Taekwondo athletes.

Causes	Youth Athletes	Collegiate Athletes	Total
Contact with Other Athletes	317 (57.6)	295 (54.4)	612 (56.0)
Inattention	67 (12.2)	35 (6.5)	102 (9.3)
Reoccurrence	48 (8.7)	41 (7.6)	89 (8.2)
Overuse	47 (8.5)	54 (10.0)	101 (9.3)
Over Training	46 (8.4)	77 (14.2)	123 (11.3)
Falling Down	8 (1.5)	6 (1.1)	14 (1.3)
Other Contact	5 (0.9)	5 (0.9)	10 (0.9)
Unstable Environment/Facility	4 (0.7)	3 (0.6)	7 (0.6)
Lack of Practice	3 (0.5)	7 (1.3)	10 (0.9)
Lack of Flexibility	2 (0.4)	8 (1.5)	10 (0.9)
Excessive Tension	2 (0.4)	0 (0)	2 (0.1)
Lack of Stamina	1 (0.2)	6 (1.1)	7 (0.6)
Other Contact	0 (0)	4 (0.7)	4 (0.3)
Lack of Practice	0 (0)	3 (0.6)	3 (0.2)
Incompleteness of the Rules	0 (0)	1 (0.2)	1 (0.1)
Total	550 (100)	542 (100)	1092 (100)

Note: Values are shown as n (%).

## Data Availability

Not applicable.
